# 1,2-Bis[4-(1*H*-imidazol-1-yl)benzyl­idene]hydrazine

**DOI:** 10.1107/S160053681202291X

**Published:** 2012-05-31

**Authors:** Yue-Bao Jin, Jie Li, Ying Zhang, Ke-Wei Lei

**Affiliations:** a State Key Lab. Base of Novel Functional Materials and Preparation Science, Institute of Solid Materials Chemistry, Faculty of Materials Science and Chemical Engineering, Ningbo University, Ningbo 315211, People’s Republic of China

## Abstract

The title compound, C_20_H_16_N_6_, is centrosymmetric with the mid-point of the N—N bond located on an inversion center. The imidazole ring is oriented at a dihedral angle of 28.03 (6)° with respect to the attached benzene ring. In the crystal, molecules are linked *via* C—H⋯N interactions.

## Related literature

For a related compound, see: Chen *et al.* (2005 ▶).
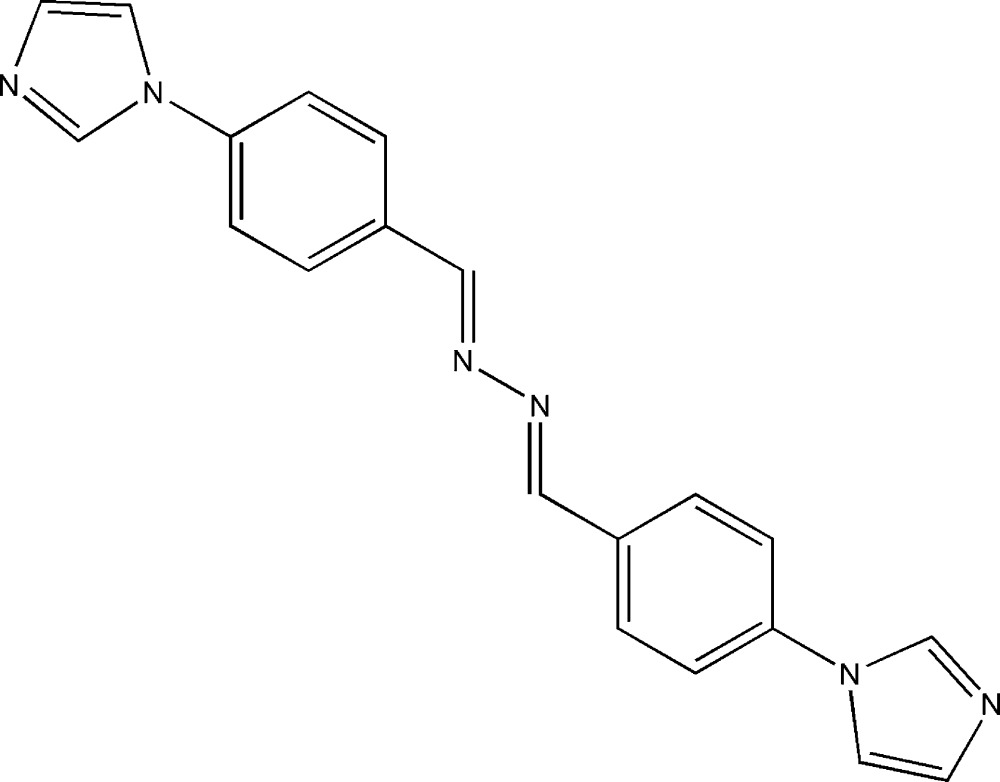


## Experimental

### 

#### Crystal data

C_20_H_16_N_6_
*M* *_r_* = 340.39
Monoclinic, 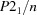

*a* = 8.1342 (3) Å
*b* = 7.8172 (3) Å
*c* = 13.2191 (6) Å
β = 90.259 (4)°
*V* = 840.55 (6) Å^3^
*Z* = 2
Mo *K*α radiation
μ = 0.09 mm^−1^
*T* = 293 K
0.44 × 0.20 × 0.15 mm

#### Data collection

Rigaku R-AXIS RAPID diffractometer
4180 measured reflections
1695 independent reflections
1249 reflections with *I* > 2σ(*I*)
*R*_int_ = 0.022

#### Refinement

*R*[*F* ^2^ > 2σ(*F* ^2^)] = 0.047
*wR*(*F* ^2^) = 0.143
*S* = 1.13
1695 reflections
118 parameters
H-atom parameters constrained
Δρ_max_ = 0.12 e Å^−3^
Δρ_min_ = −0.17 e Å^−3^

### 

Data collection: *RAPID-AUTO* (Rigaku, 1998 ▶); cell refinement: *RAPID-AUTO*; data reduction: *CrystalClear* (Rigaku/MSC, 2004 ▶); program(s) used to solve structure: *SHELXS97* (Sheldrick, 2008 ▶); program(s) used to refine structure: *SHELXL97* (Sheldrick, 2008 ▶); molecular graphics: *SHELXTL* (Sheldrick, 2008 ▶); software used to prepare material for publication: *SHELXL97*.

## Supplementary Material

Crystal structure: contains datablock(s) global, I. DOI: 10.1107/S160053681202291X/xu5536sup1.cif

Structure factors: contains datablock(s) I. DOI: 10.1107/S160053681202291X/xu5536Isup2.hkl

Supplementary material file. DOI: 10.1107/S160053681202291X/xu5536Isup3.cml

Additional supplementary materials:  crystallographic information; 3D view; checkCIF report

## Figures and Tables

**Table 1:** Hydrogen-bond geometry (Å, °)

*D*—H⋯*A*	*D*—H	H⋯*A*	*D*⋯*A*	*D*—H⋯*A*
C1—H1⋯N2^i^	0.93	2.60	3.453 (2)	153
C6—H6⋯N2^ii^	0.93	2.59	3.421 (3)	149

Symmetry codes: (i) 
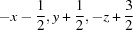
; (ii) 
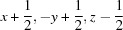
.

**Fig. 1.**
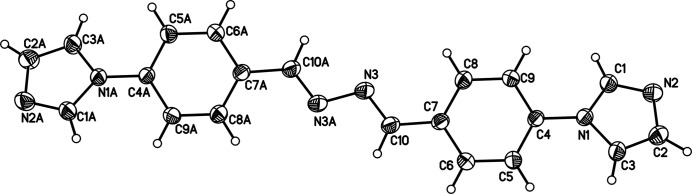
The structure of the title complex, showing 30% probability displacement ellipsoids and the atom-numbering scheme.

## supplementary crystallographic information

### Comment

The molecular structure of the title compound is illustrated in Fig. 1. The molecular structures is central symmetry. The bond lengths and bond angles in are within normal ranges. The N3—N3 bond length of 1.423 (3)%A is a slightly smaller than the normal length. The N3—C10 bond length is 1.273 (2)%A. The dihedral angle between the imidazole and benzene rings is 28.03 (6) Å

### Experimental

Hydrazine monohydrate (20 mmol, 1.08 g) and 4-imidazole benzaldehyde (40 mmol, 6.88 g) were dissolved in ethanol and the solution was refluxed for 2 h. After evaporation, a crude product was recrystallized twice from DMF and methanol to give a pure pale yellow product (Chen *et al.*, 2005). Yield: 85.2%. Calcd. for C_20_H_20_N_6_: C, 69.75; H, 5.85; N, 24.40; Found: C, 69.88; H, 5.73; N, 24.67%.

### Refinement

All H atoms were placed in geometrically idealized positions and constrained to ride on their parent atoms (C—H = 0.93 Å) and *U*_iso_(H) values equal to 1.2*U*_eq_(C).

### Crystal data

**Table:**

C_20_H_16_N_6_	*F*(000) = 356
*M**_r_* = 340.39	*D*_x_ = 1.345 Mg m^−^^3^
Monoclinic, *P*2_1_/*n*	Mo *K*α radiation, λ = 0.71073 Å
Hall symbol: -P 2yn	Cell parameters from 7835 reflections
*a* = 8.1342 (3) Å	θ = 2.9–26.4°
*b* = 7.8172 (3) Å	µ = 0.09 mm^−^^1^
*c* = 13.2191 (6) Å	*T* = 293 K
β = 90.259 (4)°	Block, yellow
*V* = 840.55 (6) Å^3^	0.44 × 0.20 × 0.15 mm
*Z* = 2	

### Data collection

**Table:**

Rigaku R-AXIS RAPID diffractometer	1249 reflections with *I* > 2σ(*I*)
Radiation source: fine-focus sealed tube	*R*_int_ = 0.022
Graphite monochromator	θ_max_ = 26.4°, θ_min_ = 2.9°
Detector resolution: 0 pixels mm^-1^	*h* = −10→8
ω scans	*k* = −9→9
4180 measured reflections	*l* = −15→16
1695 independent reflections	

### Refinement

**Table:**

Refinement on *F*^2^	Primary atom site location: structure-invariant direct methods
Least-squares matrix: full	Secondary atom site location: difference Fourier map
*R*[*F*^2^ > 2σ(*F*^2^)] = 0.047	Hydrogen site location: inferred from neighbouring sites
*wR*(*F*^2^) = 0.143	H-atom parameters constrained
*S* = 1.13	*w* = 1/[σ^2^(*F*_o_^2^) + (0.0589*P*)^2^ + 0.0922*P*] where *P* = (*F*_o_^2^ + 2*F*_c_^2^)/3
1695 reflections	(Δ/σ)_max_ < 0.001
118 parameters	Δρ_max_ = 0.12 e Å^−^^3^
0 restraints	Δρ_min_ = −0.17 e Å^−^^3^

### Special details

**Table:**

Geometry. All e.s.d.'s (except the e.s.d. in the dihedral angle between two l.s. planes) are estimated using the full covariance matrix. The cell e.s.d.'s are taken into account individually in the estimation of e.s.d.'s in distances, angles and torsion angles; correlations between e.s.d.'s in cell parameters are only used when they are defined by crystal symmetry. An approximate (isotropic) treatment of cell e.s.d.'s is used for estimating e.s.d.'s involving l.s. planes.
Refinement. Refinement of *F*^2^ against ALL reflections. The weighted *R*-factor *wR* and goodness of fit *S* are based on *F*^2^, conventional *R*-factors *R* are based on *F*, with *F* set to zero for negative *F*^2^. The threshold expression of *F*^2^ > σ(*F*^2^) is used only for calculating *R*-factors(gt) *etc*. and is not relevant to the choice of reflections for refinement. *R*-factors based on *F*^2^ are statistically about twice as large as those based on *F*, and *R*- factors based on ALL data will be even larger.

### Fractional atomic coordinates and isotropic or equivalent isotropic displacement parameters (Å^2^)

**Table:**

	*x*	*y*	*z*	*U*_iso_*/*U*_eq_	
N1	0.03400 (17)	0.26891 (18)	0.65029 (11)	0.0443 (4)	
N2	−0.1631 (2)	0.1350 (2)	0.73547 (13)	0.0605 (5)	
N3	0.44959 (19)	0.9458 (2)	0.52999 (13)	0.0559 (5)	
C1	−0.0938 (2)	0.2826 (2)	0.71607 (15)	0.0529 (5)	
H1	−0.1281	0.3856	0.7442	0.063*	
C2	−0.0742 (2)	0.0193 (3)	0.67959 (16)	0.0610 (6)	
H2	−0.0941	−0.0978	0.6783	0.073*	
C3	0.0454 (2)	0.0977 (2)	0.62695 (16)	0.0555 (5)	
H3	0.1206	0.0465	0.5836	0.067*	
C4	0.1305 (2)	0.4043 (2)	0.61009 (13)	0.0409 (4)	
C5	0.1995 (2)	0.3884 (2)	0.51484 (14)	0.0471 (5)	
H5	0.1838	0.2888	0.4775	0.057*	
C6	0.2917 (2)	0.5209 (2)	0.47552 (15)	0.0497 (5)	
H6	0.3385	0.5091	0.4118	0.060*	
C7	0.3157 (2)	0.6717 (2)	0.52950 (14)	0.0447 (5)	
C8	0.2452 (2)	0.6857 (2)	0.62521 (15)	0.0490 (5)	
H8	0.2595	0.7857	0.6623	0.059*	
C9	0.1546 (2)	0.5532 (2)	0.66558 (14)	0.0481 (5)	
H9	0.1097	0.5635	0.7299	0.058*	
C10	0.4159 (2)	0.8068 (2)	0.48435 (16)	0.0516 (5)	
H10	0.4567	0.7903	0.4195	0.062*	

### Atomic displacement parameters (Å^2^)

**Table:**

	*U*^11^	*U*^22^	*U*^33^	*U*^12^	*U*^13^	*U*^23^
N1	0.0431 (8)	0.0387 (8)	0.0513 (9)	0.0015 (6)	0.0069 (7)	0.0030 (7)
N2	0.0615 (11)	0.0520 (10)	0.0681 (12)	−0.0074 (8)	0.0195 (9)	0.0048 (8)
N3	0.0572 (10)	0.0453 (9)	0.0653 (11)	−0.0046 (8)	0.0160 (8)	0.0124 (8)
C1	0.0528 (11)	0.0464 (10)	0.0597 (13)	0.0007 (8)	0.0150 (10)	0.0014 (9)
C2	0.0690 (14)	0.0436 (11)	0.0705 (15)	−0.0099 (10)	0.0131 (11)	0.0013 (10)
C3	0.0615 (12)	0.0402 (10)	0.0650 (13)	0.0000 (9)	0.0147 (10)	−0.0029 (9)
C4	0.0393 (9)	0.0370 (9)	0.0465 (11)	0.0018 (7)	0.0028 (8)	0.0040 (7)
C5	0.0493 (11)	0.0424 (10)	0.0497 (11)	0.0009 (8)	0.0063 (9)	−0.0022 (8)
C6	0.0510 (11)	0.0492 (11)	0.0490 (12)	0.0041 (8)	0.0106 (9)	0.0033 (8)
C7	0.0402 (10)	0.0433 (10)	0.0504 (11)	0.0031 (8)	0.0032 (8)	0.0099 (8)
C8	0.0543 (11)	0.0374 (9)	0.0553 (12)	−0.0006 (8)	0.0044 (9)	−0.0001 (8)
C9	0.0554 (12)	0.0434 (10)	0.0455 (11)	−0.0004 (8)	0.0101 (9)	0.0008 (8)
C10	0.0495 (11)	0.0465 (11)	0.0590 (13)	0.0030 (8)	0.0087 (10)	0.0129 (9)

### Geometric parameters (Å, º)

**Table:**

N1—C1	1.362 (2)	C4—C9	1.389 (2)
N1—C3	1.377 (2)	C5—C6	1.382 (2)
N1—C4	1.422 (2)	C5—H5	0.9300
N2—C1	1.311 (2)	C6—C7	1.391 (3)
N2—C2	1.375 (3)	C6—H6	0.9300
N3—C10	1.273 (2)	C7—C8	1.396 (3)
N3—N3^i^	1.423 (3)	C7—C10	1.463 (2)
C1—H1	0.9300	C8—C9	1.380 (2)
C2—C3	1.346 (3)	C8—H8	0.9300
C2—H2	0.9300	C9—H9	0.9300
C3—H3	0.9300	C10—H10	0.9300
C4—C5	1.386 (2)		
			
C1—N1—C3	105.79 (15)	C6—C5—H5	120.1
C1—N1—C4	127.14 (15)	C4—C5—H5	120.1
C3—N1—C4	127.01 (16)	C5—C6—C7	121.14 (18)
C1—N2—C2	104.24 (17)	C5—C6—H6	119.4
C10—N3—N3^i^	111.6 (2)	C7—C6—H6	119.4
N2—C1—N1	112.69 (17)	C6—C7—C8	118.32 (16)
N2—C1—H1	123.7	C6—C7—C10	118.64 (18)
N1—C1—H1	123.7	C8—C7—C10	123.03 (18)
C3—C2—N2	111.20 (18)	C9—C8—C7	120.93 (17)
C3—C2—H2	124.4	C9—C8—H8	119.5
N2—C2—H2	124.4	C7—C8—H8	119.5
C2—C3—N1	106.07 (18)	C8—C9—C4	119.92 (18)
C2—C3—H3	127.0	C8—C9—H9	120.0
N1—C3—H3	127.0	C4—C9—H9	120.0
C5—C4—C9	119.88 (16)	N3—C10—C7	122.85 (19)
C5—C4—N1	119.93 (16)	N3—C10—H10	118.6
C9—C4—N1	120.19 (16)	C7—C10—H10	118.6
C6—C5—C4	119.80 (17)		
			
C2—N2—C1—N1	0.5 (2)	N1—C4—C5—C6	179.29 (15)
C3—N1—C1—N2	−0.2 (2)	C4—C5—C6—C7	−0.6 (3)
C4—N1—C1—N2	177.01 (17)	C5—C6—C7—C8	0.5 (3)
C1—N2—C2—C3	−0.6 (2)	C5—C6—C7—C10	179.37 (16)
N2—C2—C3—N1	0.5 (2)	C6—C7—C8—C9	0.3 (3)
C1—N1—C3—C2	−0.2 (2)	C10—C7—C8—C9	−178.54 (16)
C4—N1—C3—C2	−177.38 (16)	C7—C8—C9—C4	−1.0 (3)
C1—N1—C4—C5	−150.06 (18)	C5—C4—C9—C8	0.9 (3)
C3—N1—C4—C5	26.6 (3)	N1—C4—C9—C8	−178.52 (16)
C1—N1—C4—C9	29.3 (3)	N3^i^—N3—C10—C7	179.27 (17)
C3—N1—C4—C9	−154.04 (18)	C6—C7—C10—N3	−177.13 (17)
C9—C4—C5—C6	−0.1 (3)	C8—C7—C10—N3	1.7 (3)

Symmetry code: (i) −*x*+1, −*y*+2, −*z*+1.

### Hydrogen-bond geometry (Å, º)

**Table:**

*D*—H···*A*	*D*—H	H···*A*	*D*···*A*	*D*—H···*A*
C1—H1···N2^ii^	0.93	2.60	3.453 (2)	153
C6—H6···N2^iii^	0.93	2.59	3.421 (3)	149

Symmetry codes: (ii) −*x*−1/2, *y*+1/2, −*z*+3/2; (iii) *x*+1/2, −*y*+1/2, *z*−1/2.
